# Metabolomic Subtyping and Machine Learning-Based Diagnosis Reveal Clinical Heterogeneity in Silicosis

**DOI:** 10.3390/metabo16010067

**Published:** 2026-01-12

**Authors:** Jia Si, Hangju Zhu, Xinyu Ji, An-Dong Li, Ye Li, Shidan Wang, Yizhou Yang, Jianye Guo, Xinyu Li, Xiaocheng Peng, Ming Xu, Baoli Zhu, Yuanfang Chen, Lei Han

**Affiliations:** 1 Department of Science and Technology Innovation, Jiangsu Provincial Center for Disease Control and Prevention, Nanjing 210009, China; sijia@jscdc.cn (J.S.); guojianye@jscdc.cn (J.G.); sosolou@jscdc.cn (M.X.); 2 Jiangsu Province Engineering Research Center of Health Emergency, Nanjing 210009, China; zhubl@jscdc.cn; 3 Jiangsu Provincial Engineering Research Center for Disease X Organ-on-a-Chip, Nanjing 210009, China; 4 Jiangsu Cancer Hospital, Jiangsu Institute of Cancer Research, The Affiliated Cancer Hospital of Nanjing Medical University, Nanjing 210009, China; 5 School of Public Health, Nanjing Medical University, Nanjing 211166, China; 6 Department of Occupational Disease Prevention, Jiangsu Provincial Center for Disease Control and Prevention, Nanjing 210009, China; 7 School of Computer Science, Jiangsu Ocean University, Lianyungang 222005, China

**Keywords:** silicosis, untargeted metabolomics, metabolic subtypes, fatty acid metabolism, machine learning

## Abstract

Background/Objectives: Silicosis remains a major occupational health concern worldwide and is characterized by notable clinical heterogeneity in terms of disease progression and complications. However, the underlying metabolic mechanisms contributing to this heterogeneity remain poorly understood. Methods: We conducted a case–control study involving 156 silicosis patients and 132 silica-exposed controls. The plasma samples were analyzed via untargeted metabolomics based on liquid chromatography–mass spectrometry (LC-MS/MS). To explore disease subtypes and potential biomarkers, we applied non-negative matrix factorization (NMF) clustering, weighted gene co-expression network analysis (WGCNA), and machine learning approaches. Results: A total of 860 differentially abundant metabolites, including elevated pathogen-associated compounds, were identified in silicosis patients. Unsupervised NMF clustering revealed two distinct metabolic subtypes with different clinical features. Patients in the NMF2 subgroup had a 5.3-fold greater risk of pulmonary infections (*p* = 0.026) than those in the NMF1 subgroup. Metabolomic analysis revealed that NMF2 was enriched in arachidonic acid and unsaturated fatty acid metabolism pathways, with prominent LysoPC accumulation, suggesting inflammation-related lipid peroxidation. In contrast, NMF1 was characterized by increased spermidine biosynthesis and urea cycle activity, along with suppressed saturated fatty acid metabolism and reduced LysoPC processing, potentially affecting membrane integrity and promoting fibrosis. A machine learning-derived dual-metabolite panel, tyrosocholic acid and PI (20:4/0:0), achieved AUC values above 0.85 for both silicosis detection and subtype classification. Conclusions: These findings highlight metabolic heterogeneity in silicosis and suggest clinically relevant subtypes, providing a foundation for improved stratification, early detection, and targeted interventions.

## 1. Introduction

Silicosis, a progressive and irreversible lung disease caused by prolonged inhalation of silica dust, remains a significant global public health challenge. Despite regulatory advances, millions of workers worldwide continue to be affected by this functional impairment [1]. In 2019, East Asia accounted for approximately 90% of all global cases, with an age-standardized prevalence of 110.24 per 100,000 people [2], highlighting the persistent environmental and occupational health burdens in the region. In addition to causing severe pulmonary fibrosis, silicosis is associated with a significantly increased risk of complications, including lung cancer [3], and a 1.35-fold increase in tuberculosis [4]. A Chinese cohort study showed silica-exposed workers had higher mortality from hypertensive or pulmonary heart disease than unexposed individuals [5]. These systemic complications significantly worsen patient prognosis and increase mortality. Therefore, there is an urgent need for more effective and therapeutic strategies.

Nevertheless, the clinical management of silicosis is hindered by diagnostic delays and limited therapeutic options. Conventional diagnostic tools, including chest X-rays and pulmonary function tests, often fail to detect early-stage silicosis [6]. High-resolution computed tomography (HRCT) offers greater sensitivity for detecting early parenchymal changes. However, its limited accessibility and high cost hinder widespread use [7,8]. Furthermore, the incomplete understanding of silicosis pathogenesis and the lack of validated clinical classifications or therapeutic biomarkers restrict treatment to supportive care, including mechanical ventilation and lung transplantation. These limitations underscore the urgent need for innovative approaches to facilitate early detection, risk stratification, and targeted interventions for silicosis.

Metabolomics is a rapidly advancing field for elucidating disease-associated metabolic changes and identifying novel biomarkers. Unlike traditional clinical chemistry, metabolomics enables comprehensive assessment of metabolic pathways altered by environmental exposure and disease progression. In silicosis, recent studies have revealed significant alterations in arginine and proline metabolism [9,10] and disruptions in lipid metabolism, including elevated ceramide levels linked to chronic inflammation and fibrosis [11]. These findings suggest that metabolomics provides valuable mechanistic insights into silicosis pathogenesis and facilitates the development of standardized diagnostic tools for improved early detection strategies [12].

To address the current gaps in silicosis management, this study leverages metabolomics to improve disease classification and risk prediction. We investigated patient heterogeneity through metabolomic profiling and revealed metabolic variations that potentially underlie different disease subtypes. We seek to identify robust biomarkers and metabolic pathways that distinguish silicosis patients from healthy individuals, predict disease severity, and identify individuals at increased risk of complications. Ultimately, this research aims to identify novel classification criteria to distinguish silicosis patients, improve diagnostic prediction, inform personalized treatment strategies, and mitigate the burden of devastating environment-linked silicosis.

## 2. Materials and Methods

### 2.1. Sample Collection

We conducted a case–control study with 156 silicosis patients and 132 occupationally silica-exposed controls from pneumoconiosis rehabilitation facilities across Jiangsu Province, China, between January and December 2023. Silicosis was diagnosed according to the Chinese national standard GBZ 70-2015 Diagnostic Criteria of Occupational Pneumoconiosis [13], and all cases had a documented history of occupational silica exposure. Controls were defined as silica-exposed workers without clinical, radiographic, or functional evidence of occupational lung diseases. Cases and controls were matched on key variables, including sex, occupational category, employment duration, cumulative silica exposure levels, and age (±5 years).

### 2.2. Plasma Extraction and LC-MS/MS-Based Untargeted Metabolomics

Fasting whole blood samples (2.0 mL) were collected and centrifuged at 3000 rpm for 10 min at 4 °C to isolate plasma. For metabolomic analysis, 100 μL plasma aliquots were transferred to 1.5 mL Eppendorf tubes (Eppendorf, Hamburg, Germany) and mixed with 400 μL ice-cold protein precipitation solvent (acetonitrile: methanol, 3:1 *v*/*v*, Sigma-Aldrich, St. Louis, MO, USA). Samples were vortexed (30 s), sonicated in an ice bath (10 min), and incubated at −20 °C for 1 h. Following centrifugation (12,000 rpm, 10 min, 4 °C), the supernatants were collected and evaporated to dryness using a vacuum concentrator at 4 °C. The residue was reconstituted in 100 μL of 40% acetonitrile (Sigma-Aldrich, St. Louis, MO, USA), vortexed (30 s), and centrifuged again (12,000 rpm, 10 min, 4 °C). The final supernatants were analyzed by LC-MS/MS.

Quality control (QC) samples were prepared by pooling 10 μL aliquots from each individual plasma sample to represent the total metabolic profile of the entire cohort. Untargeted metabolomics was performed using liquid chromatography–tandem mass spectrometry (LC-MS/MS) on a Orbitrap Exploris 240 mass spectrometer coupled to a Vanquish UHPLC system (Thermo Fisher Scientific, Waltham, MA, USA). At the beginning of the analytical sequence, five QC samples were injected to equilibrate the column and instrument, followed by the injection of one QC sample every 10 experimental samples throughout the run. Solvent and method blanks were also injected periodically to monitor potential background noise and carryover. Separation was achieved on a C18 reversed-phase column (Waters ACQUITY UPLC HSS T3, 2.1 × 100 mm, 1.8 μm; Waters Corporation, Milford, MA, USA; flow rate: 0.3 mL/min; column temperature: 40 °C). Gradient elution used mobile phases of (A) 0.1% formic acid in water (positive mode) or 0.1% ammonium hydroxide/20 mM ammonium acetate in water (negative mode) and (B) acetonitrile. The injection volume was 5 μL. Detailed gradient parameters are provided in Table S1.

### 2.3. Data Preprocessing

The raw LC-MS/MS data were processed using the XCMS (3.12.0, Scripps Research Institute, La Jolla, CA, USA). Peak picking was performed via the CentWave algorithm with a mass tolerance of 10 ppm, peak width of 5–30 s, and a signal-to-noise ratio threshold (snthresh) of 3. Metabolite identification was conducted using MetDNA2 (1.4.5.92, University of Texas at Arlington, Arlington, TX, USA), applying a mass tolerance of 15 ppm for MS1 and 25 ppm for MS2 fragments, with a retention time tolerance of 60 s. To ensure high-confidence identification, a minimum of 4 matched MS2 fragments was required for each feature.

The data preprocessing pipeline: (1) Features exhibiting >20% missing values in either biological samples or QC samples were excluded. The missing values were imputed using the k-nearest neighbor (KNN) algorithm with default parameters. (2) Normalization and transformation were performed using log10 transformation for variance stabilization. (3) The Relative Standard Deviation (RSD) was calculated as the standard deviation divided by the mean of peak intensities across QC samples. Features with an RSD > 20% in QC samples were strictly excluded. Principal component analysis (PCA) was performed via the “prcomp” function in R to visualize sample clustering and identify potential outliers. All the statistical analyses were performed in R (version 4.4.1; R Foundation for Statistical Computing, Vienna, Austria).

### 2.4. Identification of Differentially Abundant Metabolites (DAMs)

Partial least squares discriminant analysis (PLS-DA) was conducted using the “ropls” package (1.36.0) to generate variable importance in projection (VIP) scores, which were used to quantify metabolite contributions to group discrimination. For pairwise comparisons, parametric analysis of variance (ANOVA) was applied to homoscedastic metabolites (assessed by Levene’s test), whereas the non-parametric Mann–Whitney U test was used for heteroscedastic metabolites. DAMs were identified on the basis of a false discovery rate (FDR)-adjusted *p* < 0.05 and a VIP > 1. Subsequently, pathway enrichment analysis of DAMs was performed via MetaboAnalyst 6.0 (https://www.metaboanalyst.ca/, accessed on 9 January 2025).

### 2.5. Metabolic Subtyping and Factor Analysis

We employed non-negative matrix factorization (NMF, 0.28) to delineate metabolic heterogeneity within the silicosis cohort. The optimal number of clusters (k) was determined by iteratively applying NMF for k values from 2 to 10. The optimal k value was selected on the basis of the maximum cophenetic correlation coefficient and the elbow point of the residual curve. Subtype-specific metabolites were identified using the “extractFeatures” function and subsequently employed to classify patients into distinct metabolic subtypes.

### 2.6. Weighted Co-Expression Network Analysis

Weighted co-expression networks were constructed using the “WGCNA” package (1.73) in R, based on metabolomic profiles from healthy controls and NMF-classified patient subtypes. The optimal soft-thresholding power was selected to construct a scale-free adjacent matrix, which was then transformed into a topological overlap matrix (TOM). Modules were identified via hierarchical clustering of the 1-TOM dissimilarity matrix, followed by dynamic tree cutting. The first principal component of each module was extracted and defined as its module eigengene (ME), which represents the module’s overall expression profile. Significant MEs were retained for downstream analysis via Pearson correlation analysis with patient subtypes (|r| > 0.8, *p* < 0.05).

### 2.7. Biomarker Discovery Through Machine Learning

We developed a robust diagnostic framework for silicosis subtyping through an integrated computational and biological validation pipeline. This approach synergistically incorporated LASSO regression, the Boruta algorithm, and multivariate unbiased variable selection in PLS analysis (MUVR-PLS) to identify stable biomarkers, retaining those that were consistently selected across all three methods. Candidate biomarkers then underwent rigorous biological validation, including strict quality controls: intragroup coefficient of variation <10% and significant intergroup median expression difference >0.6.

Following initial screening, the XGBoost algorithm was applied for further feature selection and optimization of the final metabolite panel. The XGBoost classifier was developed using stratified 70/30 data splitting and 10-fold cross-validation to ensure model robustness and generalizability. This final model achieved high diagnostic accuracy, with area under the receiver operating characteristic curve (AUC) values assessed in independent validation cohorts.

## 3. Results

### 3.1. Dysregulated Host Lipid Metabolism and Pathogen-Associated Metabolites in Silicosis

**Figure 1:**
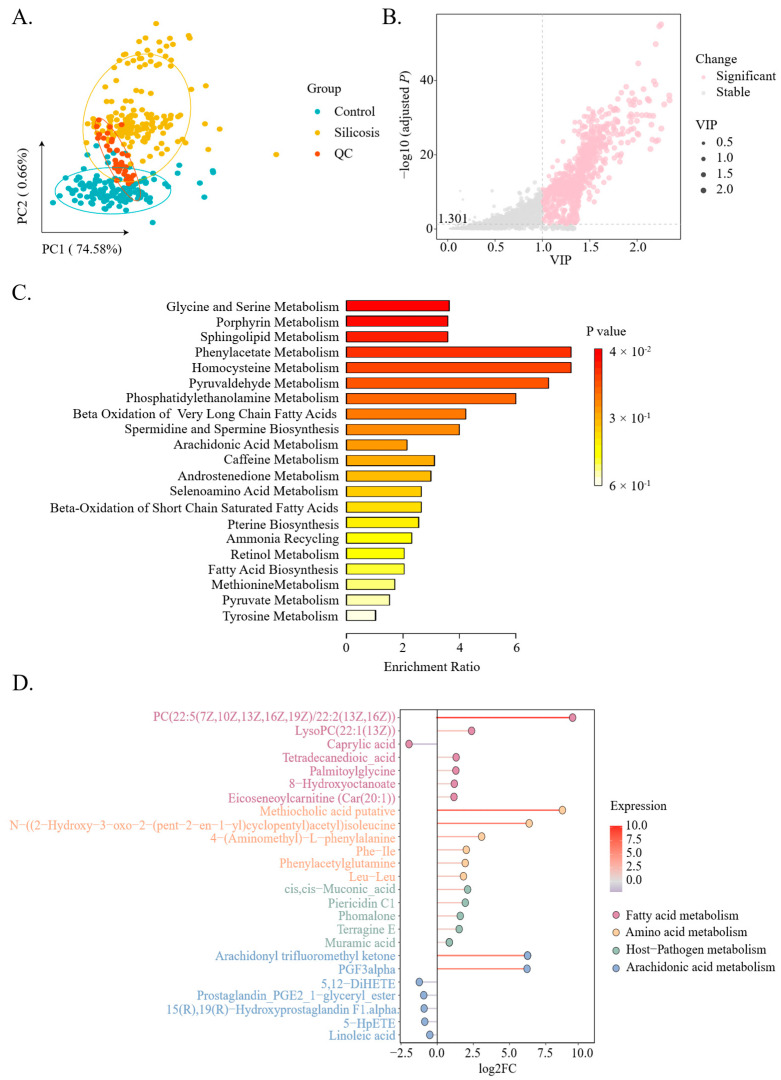
Metabolomic profiling of silicosis based on untargeted LC-MS/MS analysis. (**A**) Principal component analysis (PCA) plot demonstrating the distinct metabolomic profiles of silicosis patients, healthy controls, and quality control (QC) samples. (**B**) Scatter plot illustrating metabolite differences between silicosis patients and healthy controls, with pink dots highlighting statistically significant changes (adjusted-*p* < 0.05, VIP > 1). (**C**) Bar plot illustrating enriched metabolic pathways associated with differentially abundant metabolites (DAMs). (**D**) Lollipop chart comparing the relative abundance levels of representative DAMs between silicosis patients and healthy controls.

Untargeted metabolomic profiling was conducted on plasma samples from a cohort of 156 silicosis patients and 132 matched controls, yielding 2177 high-quality metabolites (Figure S1). Principal component analysis (PCA) revealed distinct metabolic clustering between groups (Figure 1A), confirming analytical robustness. The baseline characteristics of the study population are summarized in Table S2.

Combined PLS-DA and univariate analysis revealed 860 differentially abundant metabolites (DAMs) distinguishing silicosis patients from controls (Figure 1B), with significant enrichment in glycine and serine metabolism (L-serine and 5-aminolevulinate), sphingolipid metabolism (sphingosine), β-oxidation of very long chain fatty acids (caprylic and capric acids), spermidine and spermine biosynthesis (S-adenosylmethioninamine), and arachidonic acid metabolism (5,12-DiHETE and 5-HpETE) (Figure 1C,D). Notably, patients with silicosis presented elevated plasma levels of pathogen-associated metabolites, including fungal-derived compounds (phomalone), bacterial-origin metabolites (piericidin C1 and muramic acid), and microbiota-modified aromatic metabolites (cis, cis-muconic acid and terragine E) (Figure 1D). These findings suggest that chronic silica exposure may contribute to secondary microbial dysbiosis or disrupt host–pathogen interactions.

### 3.2. Metabolic Subtyping Revealed Differential Complication Risks in Silicosis

**Figure 2:**
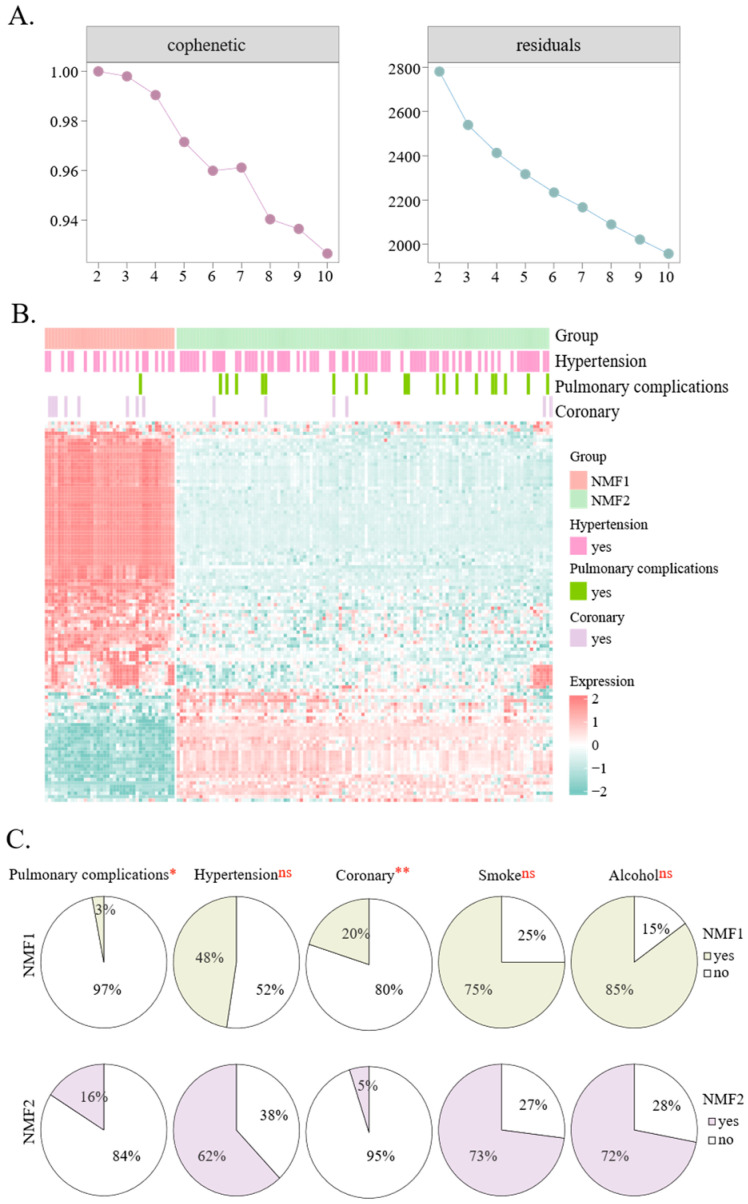
Metabolomics and comorbidity heterogeneity in silicosis subtypes. (**A**) Cophenetic and residual curves of non-negative matrix factorization (NMF) clustering at rank = 2, demonstrating optimal subtype separation. (**B**) Heatmap illustrating distinct metabolomic profiles of patient subtypes derived from NMF analysis. (**C**) Pie charts illustrating the prevalence of comorbidities and sociodemographic characteristics between the NMF1 and NMF2 subtypes. *p* < 0.05 is indicated by a single asterisk (*), *p* < 0.01 by a double asterisk (**), and ns denotes not significant.

Non-negative matrix factorization (NMF) clustering revealed substantial metabolic heterogeneity among silicosis patients. The optimal rank was determined to be K = 2 based on consensus map inspection and clustering stability (Figure 2A, Figures S2 and S3), effectively stratifying them into two distinct subtypes: NMF1 (*n* = 40) and NMF2 (*n* = 116) (Figure 2B). These subtype groups presented similar sociodemographic characteristics, including smoking history, alcohol consumption, and body mass index (BMI). However, their comorbidity patterns differed markedly. While the incidence of hypertension was high in both subtypes (48% in NMF1 vs. 62% in NMF2), the NMF2 subtype demonstrated a significantly higher susceptibility to pulmonary complications. Quantitative analysis revealed that the NMF2 subgroup had a 5.3-fold greater incidence of pulmonary complications than the NMF1 subgroup did (Fisher’s exact test, *p* = 0.026; Figure 2C), which was further supported by the results of logistic regression analysis (Table S3).

### 3.3. Distinct Inflammatory and Fibrogenic Metabolic Dysregulation in the NMF2 and NMF1 Subtypes

**Figure 3:**
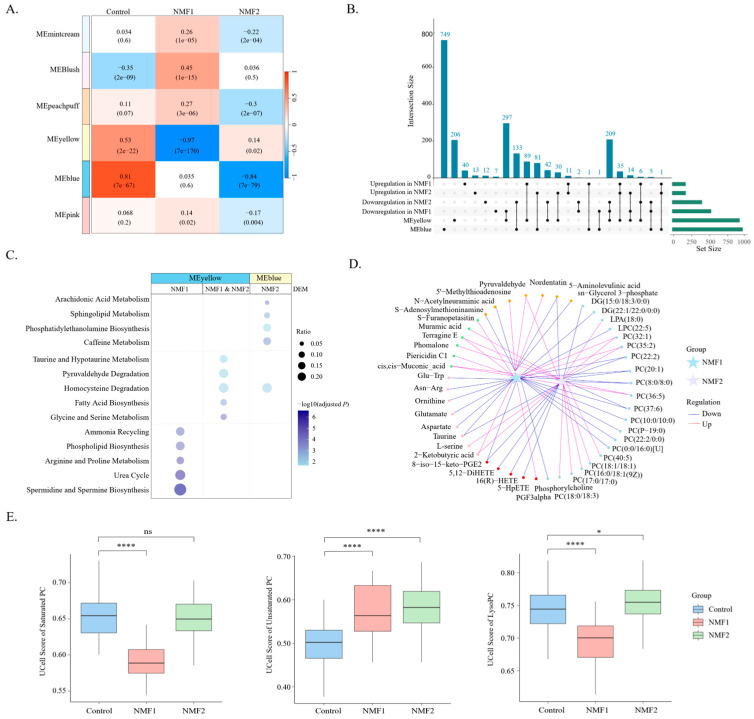
WGCNA of metabolic dysregulation in silicosis subtypes. (**A**) Heatmap illustrating the correlation between WGCNA-derived modules and the control, NMF1, and NMF2 phenotypes. (**B**) UpSetR plot demonstrating the overlap of metabolites in silicosis-related functional modules and differentially abundant metabolites (DAMs) in the NMF1 and NMF2 compared with the control. (**C**) Dot plot illustrating enriched metabolic pathways across different modules and subtypes. (**D**) Representative altered metabolites in NMF1 and NMF2 subtypes. (**E**) Box plots depicting signature scores of saturated PC, unsaturated PC, and LysoPC among the control, NMF1, and NMF2 groups. ****, *p* < 0.001. *, *p* < 0.05. ns, not significant.

Weighted gene co-expression network analysis (WGCNA) identified two key metabolite modules associated with silicosis phenotypes (Figure 3A). The blue module showed a strong inverse correlation with the NMF2 subtype (Pearson’s r = −0.84, *p* < 0.001). Notably, a total of 36.97% (220/595) of NMF2-specific metabolites clustered within this module, in contrast to only 1.11% (8/718) in NMF1 (χ^2^ = 289.10, *p* < 0.001) (Figure 3B). Pathway analysis revealed that these NMF2-specific metabolites were predominantly enriched in pro-inflammatory processes, including sphingolipid metabolism (CerP (d18:1/12:0)) and arachidonic acid metabolism (16 (R)-HETE, 5-HpETE) (Figure 3C,D, Figures S4 and S5).

Conversely, the yellow module demonstrated a predominant association with fibrotic progression in NMF1 (Pearson’s r = 0.97, *p* < 0.001), encompassing 90.53% (650/718) of this subtype’s differentially abundant metabolites versus 56.47% (336/595) in NMF2 (χ^2^ = 199.99, *p* < 0.001) (Figure 3A,B). The NMF1 subtype exhibited a distinctive fibrosis-promoting profile and was enriched in spermidine and spermine biosynthesis (S-Adenosylmethioninamine and 5′-methylthioadenosine), urea cycle (L-glutamic acid and L-aspartic acid), and arginine and proline metabolism (ornithine) (Figure 3C,D). Although both subtypes exhibited phosphatidylcholine (PC) metabolism disorders, Ucell analysis revealed significant subtype-specific differences in their pathological mechanisms (Figure 3E). The NMF1 subgroup is characterized predominantly by reduced saturated fatty acid metabolism and decreased LysoPC processing. These metabolic alterations may compromise cellular membrane stability, leading to fibrogenic remodeling. Conversely, the NMF2 subtype presented increased unsaturated fatty acid metabolism with LysoPC accumulation. This metabolic profile likely promotes lipid peroxidation and triggers oxidative stress-mediated inflammation. The observed associations between metabolism and silicosis subtypes indicate that the NMF1 subgroup is characterized primarily by fibrotic processes, whereas NMF2 is driven mainly by inflammation.

### 3.4. Dual-Metabolite Biomarkers for Accurate Silicosis Subtype Diagnosis

**Figure 4:**
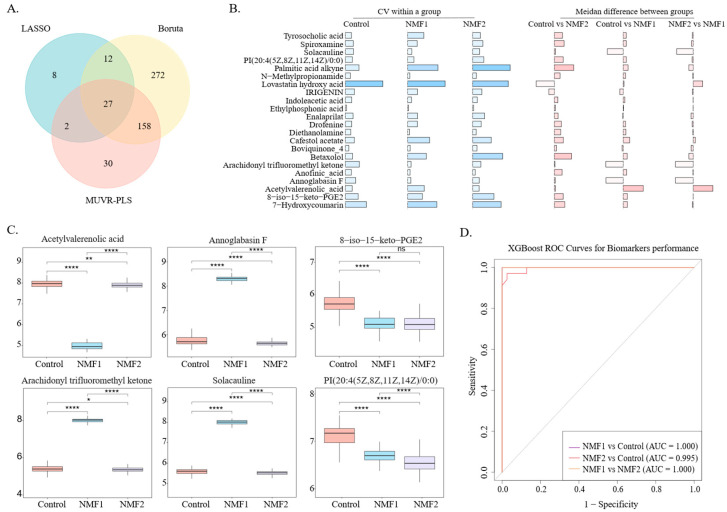
Identification of potential biomarkers for silicosis subtypes. (**A**) Venn diagram illustrating the overlap of metabolites identified by the LASSO, Boruta, and MUVR-PLS feature selection methods. (**B**) Bar plot depicting the intragroup coefficients of variation (left, blue) and intergroup median expression differences (right, pink) for the identified metabolites. (**C**) Box plot showing the relative expression levels of potential biomarkers across the control, NMF1, and NMF2 groups. (**D**) Receiver operating characteristic (ROC) curves demonstrating the diagnostic capability of tyrosocholic acid and PI (20:4/0:0) in differentiating silicosis subtypes and controls. *p* < 0.05 is indicated by a single asterisk (*), *p* < 0.01 by a double asterisk (**), *p* < 0.0001 by a quadruple asterisk (****), and ns denotes not significant.

We developed a clinically applicable diagnostic model for silicosis subtyping through a multi-stage screening and validation pipeline. This pipeline was able to identify 27 candidate metabolites (Figure 4A, Table S4) and refine them to six potential biomarkers (Figure 4B). Quantitative analysis revealed consistent reductions in 8-iso-15-keto-PGE2 and PI (20:4/0:0) levels across both NMF1 and NMF2 subtypes compared to controls, with acetylvalerenolic acid specifically reduced in NMF1 (Figure 4C). In contrast, NMF1 showed significantly elevated levels of solacauline, tyrosocholic acid, and annoglabasin F, which could be subtype-specific candidate biomarkers. The XGBoost algorithm revealed that the combination of tyrosocholic acid and PI (20:4/0:0) provided optimal diagnostic performance. External validation confirmed that these two metabolites exhibited superior diagnostic performance, with AUC values exceeding 0.85 for distinguishing between silicosis patients and healthy controls, as well as for classifying silicosis subtypes (Figure 4D).

## 4. Discussion

This study identified two clinically significant metabolic subtypes (NMF1 and NMF2) in silicosis through comprehensive metabolic analysis, revealing distinct pathway alterations associated with disease variation and progression. These findings highlight the critical roles of lipid dysregulation and host–microbe metabolic interactions in the pathogenesis of silicosis. The identification of subtype-specific mechanisms and biomarkers has important implications for advancing personalized medicine in silicosis management.

### 4.1. Metabolic Divergence and Fibrotic Mechanisms

The NMF1 subtype is characterized by significant dysfunction in the urea cycle and impaired polyamine synthesis, particularly marked by a reduction in ornithine and S-adenosylmethioninamine (SAM). These deficiencies jointly contribute to a microenvironment conducive to fibrogenesis. As a core urea cycle intermediate [14], ornithine deficiency disrupts citrulline and arginine synthesis, impairing ammonia detoxification and resulting in cytotoxic hyperammonemia with subsequent mitochondrial dysfunction. Concurrently, the simultaneous depletion of ornithine and SAM, both crucial precursors for spermidine and spermine biosynthesis, undermines polyamine-mediated cytoprotection, resulting in impaired epithelial autophagy and disrupted redox homeostasis [15,16]. This interconnected metabolic failure resembles the TGF-β1-driven fibrotic reprogramming observed in idiopathic pulmonary fibrosis (IPF) [17], where chronic oxidative stress and ammonia toxicity converge to induce alveolar epithelial cell senescence and promote the secretion of pro-fibrotic cytokines. In addition, NMF1 notably depleted saturated phosphatidylcholines (PCs). The chronic loss of saturated PCs destabilizes lipid rafts [18], which are critical for TGF-β receptor signaling [19], thereby promoting fibrotic patterning. Collectively, these metabolic abnormalities in NMF1 promote a fibrotic microenvironment, highlighting the intricate link between metabolic dysfunction and fibrotic disease progression.

In contrast, NMF2 is characterized by pronounced dysregulation of arachidonic acid metabolism. While previous studies have reported abnormalities in the COX pathway [20], our research revealed additional disturbances in the CYP450 and LOX pathways in silicosis. Specifically, we observed a significant reduction in 16(R)-HETE, 5-HpETE, and 5,12-DiHETE, which are crucial for modulating immune responses. These metabolites play vital roles in regulating the adhesion and aggregation of polymorphonuclear leukocytes [21], reactive oxygen species (ROS) activity, and the release of pro-inflammatory cytokines such as TNF-α, IL-1, and IL-6 [22]. This metabolic imbalance may contribute to persistent inflammation and progressive tissue damage. The observed lipidomic alterations further support these pathological abnormalities. Elevated polyunsaturated PCs fuel lipid peroxidation cascades, generating mitochondrial ROS and triggering ferroptotic cell death [23]. Concurrently, elevated LysoPC can polarize macrophages toward pro-inflammatory M1 phenotypes and promote NADPH oxidase-dependent superoxide production [24]. These synergistic effects establish a vicious cycle in which persistent inflammation and membrane peroxidation compromise pulmonary barrier integrity, whereas impaired antimicrobial defenses facilitate microbial colonization. These abnormalities may explain the 5.3-fold increased risk of pulmonary complications observed in NMF2 patients. Collectively, these results underscore the complex interplay between lipid metabolism, oxidative stress, and immune dysfunction in NMF2-type silicosis, providing new insights into disease pathogenesis and potential therapeutic targets.

### 4.2. Microbial Metabolic Crosstalk: A Novel Dimension

This study reveals a novel triad involved in silicosis pathogenesis, comprising silica exposure, pulmonary dysbiosis, and metabolic reprogramming. Both the NMF1 and NMF2 subtypes exhibited significant enrichment of pathogen-derived metabolites, including bacterial-specific markers (muramic acid) and microbiota-modified aromatic compounds (cis, cis-muconic acid and terragine E). These findings suggest that silica-induced epithelial barrier disruption facilitates pulmonary microbial imbalance, leading to the accumulation of bioactive microbial metabolites that synergistically interact with host metabolic dysfunction. These observations are consistent with recent 16S rRNA sequencing data from pneumoconiosis cohorts, which demonstrated a predominance of firmicutes, bacteroidota and proteobacteria [25]. The accumulated microbial metabolites potentially serve dual roles as both diagnostic biomarkers and functional mediators in silicosis pathobiology.

Microbial metabolites can modulate host biological responses through immunomodulatory pathways that potentially exacerbate inflammatory and fibrotic processes. For example, muramic acid, a component of bacterial cell walls [26], activates innate immune receptors (TLR2/4) [27], thereby promoting neutrophil infiltration and initiating inflammatory cascades. Similarly, cis, cis-muconic acid, which is produced through aromatic compound degradation by Pseudomonas species [28], may directly impair mitochondrial function in alveolar epithelial cells [29]. Furthermore, microbial dysbiosis modulates host disease phenotypes through perturbations in amino acid metabolism. Notably, microbial competitive utilization of glutamic and aspartic acids may deplete substrates essential for urea cycle completion and glutathione biosynthesis. This depletion could establish a feed-forward loop between dysbiosis and epithelial failure.

### 4.3. Translational Implications of Biomarkers

The joint identification of phosphatidylinositol (PI) and tyrosocholic acid as complementary biomarkers represents a promising translational opportunity in silicosis research. PI, a key phospholipid involved in membrane trafficking and intracellular signal transduction, is highly enriched in the endoplasmic reticulum (ER) and plasma membranes and participates in phosphoinositide-dependent stress signaling pathways, including the PI3K/Akt/mTOR cascades, which are implicated in silica-induced macrophage activation and fibrosis progression [30,31]. Depletion of PI may therefore reflect silica-induced ER stress, membrane remodeling, and early disruption of lipid homeostasis at the cellular level. In parallel, tyrosocholic acid, a bile acid-related lipid metabolite, integrates host lipid metabolism with inflammatory and immune responses in the lung microenvironment. Bile acids and their derivatives have been shown to signal through receptors such as FXR and TGR5 to modulate oxidative stress, macrophage activation, and tissue remodeling in chronic inflammatory and fibrotic diseases [32]. These two metabolites thus report distinct yet converging aspects of lipid dysregulation; PI reflects early membrane/ER and phosphoinositide-stress signaling, and tyrosocholic acid reflects downstream inflammatory–metabolic imbalance along the bile acid–immune axis. A combined signature of reduced PI together with elevated tyrosocholic acid could flag patients at risk of rapid fibro-inflammatory progression and enable non-invasive stratification of silicosis subtypes using serum or bronchoalveolar lavage fluid, thereby informing more refined monitoring strategies. This, in turn, may provide a rationale for exploring lipid-targeted interventions aimed at restoring membrane integrity and bile acid-related metabolic homeostasis in silicosis.

### 4.4. Study Limitations

While this investigation provides novel insights into the metabolic landscape of silicosis and establishes clinically relevant disease subtypes with biomarker signatures, several critical limitations necessitate consideration: (1) The cross-sectional design limits formal causal inference between metabolic alterations and disease progression. Further mechanistic studies, including animal models and functional assays, are essential to validate these findings and elucidate the underlying pathways involved in silicosis pathogenesis. (2) Although metabolomics-based subtypes and associated biomarkers show promise, their clinical utility, such as in disease risk prediction or personalized treatment strategies, requires multicenter validation. (3) The role of the lung microbiome in silicosis remains poorly understood and warrants further investigation. The complex interactions between the microbiome and host metabolism may offer novel therapeutic targets for silicosis. (4) Lastly, while silica-exposed controls minimize occupational confounders, potential subclinical changes in this group could “blur” group distinctions. Despite the clear segregation observed in our current data, future validation using a three-tier cohort (unexposed, exposed-healthy, and diseased) is necessary to further refine the specificity of these pathogenic biomarkers.

## 5. Conclusions

In summary, this study provides a comprehensive characterization of metabolic heterogeneity in silicosis. We propose a novel classification of silicosis subtypes and identify NMF2 as a critical subpopulation associated with an increased risk of complications. Our findings highlight the significant roles of fatty acid metabolism, polyamine metabolism, and arachidonic acid metabolism in the pathogenesis and heterogeneous progression of silicosis. Additionally, we identified potential biomarkers that may guide clinical practice. These insights into the metabolic landscape of silicosis pave the way for future diagnostic and therapeutic strategies.

## Data Availability

The raw data supporting the conclusions of this article will be made available by the authors on request.

## Supplementary Materials

The following supporting information can be downloaded at https://www.mdpi.com/article/10.3390/metabo16010067/s1, Figure S1: Sample Design and Metabolomics Data Quality Overview; Figure S2: Evaluation of NMF clustering stability and rank selection; Figure S3: PCA of NMF subtype clustering and comorbidity effects; Figure S4: Enrichment Analysis of Differentially Expressed Genes for Silicosis Subtypes; Figure S5: Metabolite Type Distribution and Regulatory Networks for Key Metabolites in Silicosis Subtypes; Table S1: Liquid Chromatography Gradient Elution Conditions; Table S2: Baseline Characteristics of the Cohort; Table S3: Results of the Logistic Regression Model; Table S4: Expression Levels of 27 Selected Metabolites Across Silicosis Subtypes and Healthy Controls.

## Abbreviations

The following abbreviations are used in this manuscript:

NMF	non-negative matrix factorization
HRCT	high-resolution computed tomography
LC-MS/MS	liquid chromatography–tandem mass spectrometry
ME	module eigengene
AUC	area under the receiver operating characteristic curve
WGCNA	weighted gene co-expression network analysis
DAM	differentially abundant metabolites
FDR	false discovery rate
VIP	importance in projection
PLS-DA	partial least squares discriminant analysis
MUVR-PLS	multivariate unbiased variable selection in PLS analysis
ANOVA	analysis of variance
LC-MS	liquid chromatography–mass spectrometry
KNN	k-nearest neighbor
RSD	relative standard deviation
