# Supplementary material for: Metabolomic Subtyping and Machine Learning-Based Diagnosis Reveal Clinical Heterogeneity in Silicosis
Source: Metabolites. 2026 Jan 12;16(1):67. doi: 10.3390/metabo16010067 (PMC12843767; doi:10.3390/metabo16010067)
Supplement: Supplementary file 1 [file metabolites-16-00067-s001.zip › metabolites-4067204-supplementary.pdf]

Supplementary Data

Table S1 Liquid Chromatography Gradient Elution Conditions

Table S2. Baseline Characteristics of the Cohort

Table S3. Results of the Logistic Regression Model

Figure S1. Sample Design and Metabolomics Data Quality Overview

Figure S2. Evaluation of NMF clustering stability and rank selection

Figure S3. PCA analysis of NMF subtype clustering and comorbidity effects

Figure S4. Enrichment Analysis of Differentially Expressed Genes for Silicosis Subtypes

Figure S5. Metabolite Type Distribution and Regulatory Networks for Key Metabolites in Silicosis Subtypes

Table S4. Expression Levels of 27 Selected Metabolites Across Silicosis Subtypes and Healthy Controls

Table S1. Liquid Chromatography Gradient Elution Conditions

Time（min）	A（%）	B（%）
0	98	2
0.5	98	2
18	5	95
21	5	95
21.1	98	2
25	98	2

The ion source was electrospray ionization (ESI), with a positive ion voltage set at 3400 V and a negative ion voltage at -2000 V. The gas mode was static, with sheath gas set to 40, auxiliary gas at 10, and sweep gas at 0. The ion transfer tube temperature was maintained at 350℃, while the vaporizer temperature was set to 400℃. Data acquisition was performed in a data-dependent mode, with 10 dependent scans.

Table S2. Baseline Characteristics of the Cohort

	Control（N=132）	Silicosis（N=156）	*P*
Age（y）	68.65±5.76	69.31±6.66	0.376
Sex: Male	132 (100%)	156 (100%)	-
Smoke:			0.943
Never	37 (28.0%)	41 (26.3%)	
Former	49 (37.1%)	60 (38.5%)	
Current	46 (34.8%)	55 (35.3%)	
Alcohol:			0.521
Never	27 (20.5%)	38 (24.4%)	
Former	43 (32.6%)	42 (26.9%)	
Current	62 (47.0%)	76 (48.7%)	
CDE（mg/m^3-years）	1505.12±397.37	1564.50±411.59	0.216
BMI（kg/m^2）	25.25±2.59	24.17±3.00	0.001

Table S3. Results of the Logistic Regression Model

Variable	*b*	S_b	Wald x^2	*P*	OR (95%CI)
Age of diagnosis	-0.139	0.027	-5.217	<0.001***	0.870(0.821, 0.913)
BMI	-1.421	0.805	-1.765	0.078	0.241(0.047, 1.160)
Pulmonary complications†	2.527	1.153	2.191	0.028*	12.521(1.953, 261.452)
Hypertension	1.019	0.481	2.117	0.034*	2.771(1.096, 7.360)

†Pulmonary complications: pneumonia, trachitis, bronchitis, COPD, pulmonary heart

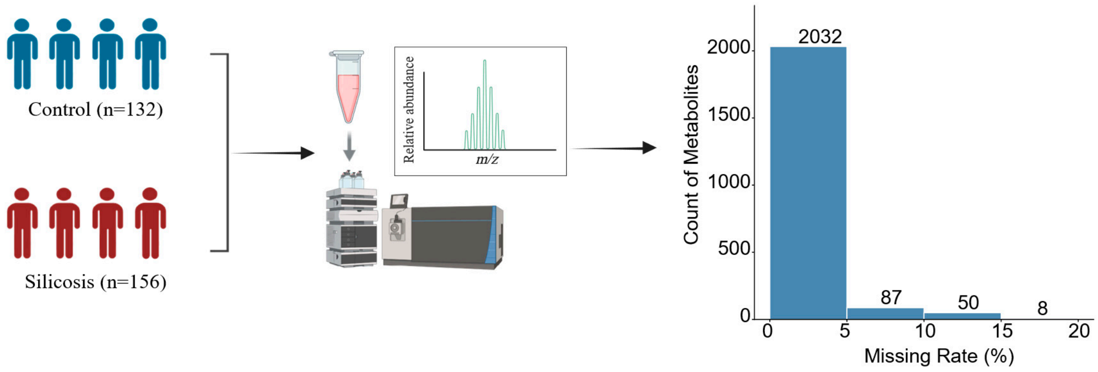

Figure S1. Sample Design and Metabolomics Data Quality Overview.

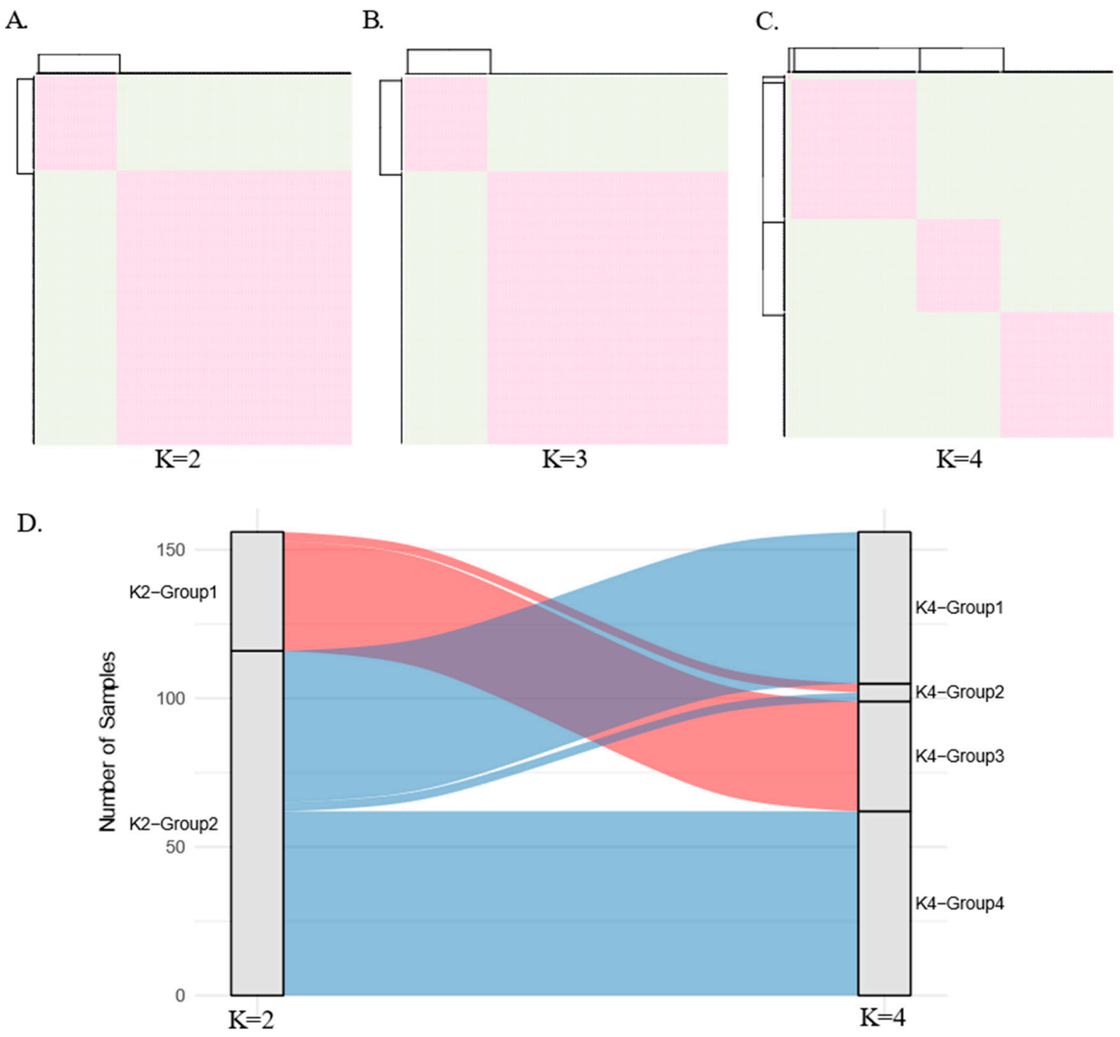

Figure S2. Evaluation of NMF clustering stability and rank selection.

(A) Consensus map for K=2. (B). Consensus map for K=3. The heatmap shows that the samples do not separate into three distinct, stable blocks, indicating that a 3-cluster solution is mathematically unstable for this dataset. (C) Consensus map for K=4. The samples are clustered into four groups. (D) Sankey diagram illustrating the relationship between K=2 and K=4 clustering assignments.

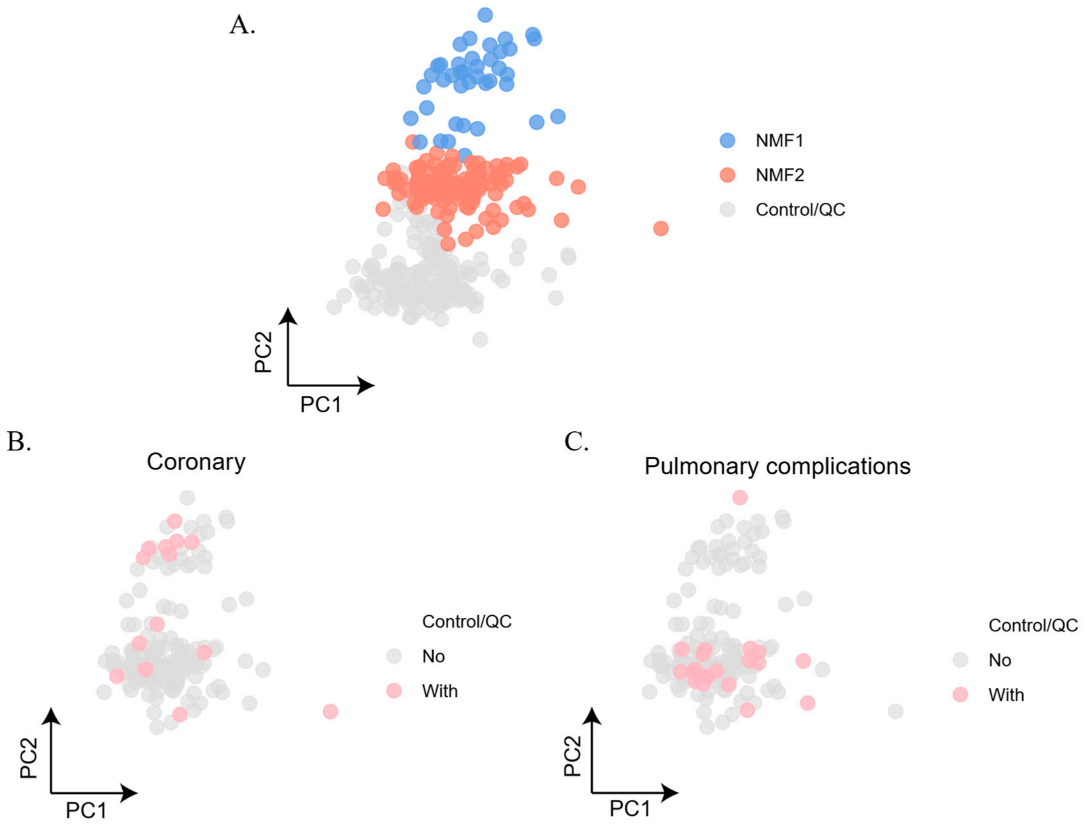

Figure S3. PCA analysis of NMF subtype clustering and comorbidity effects.
(A) NMF subtypes. (B-C) Overlay of coronary and pulmonary complications.

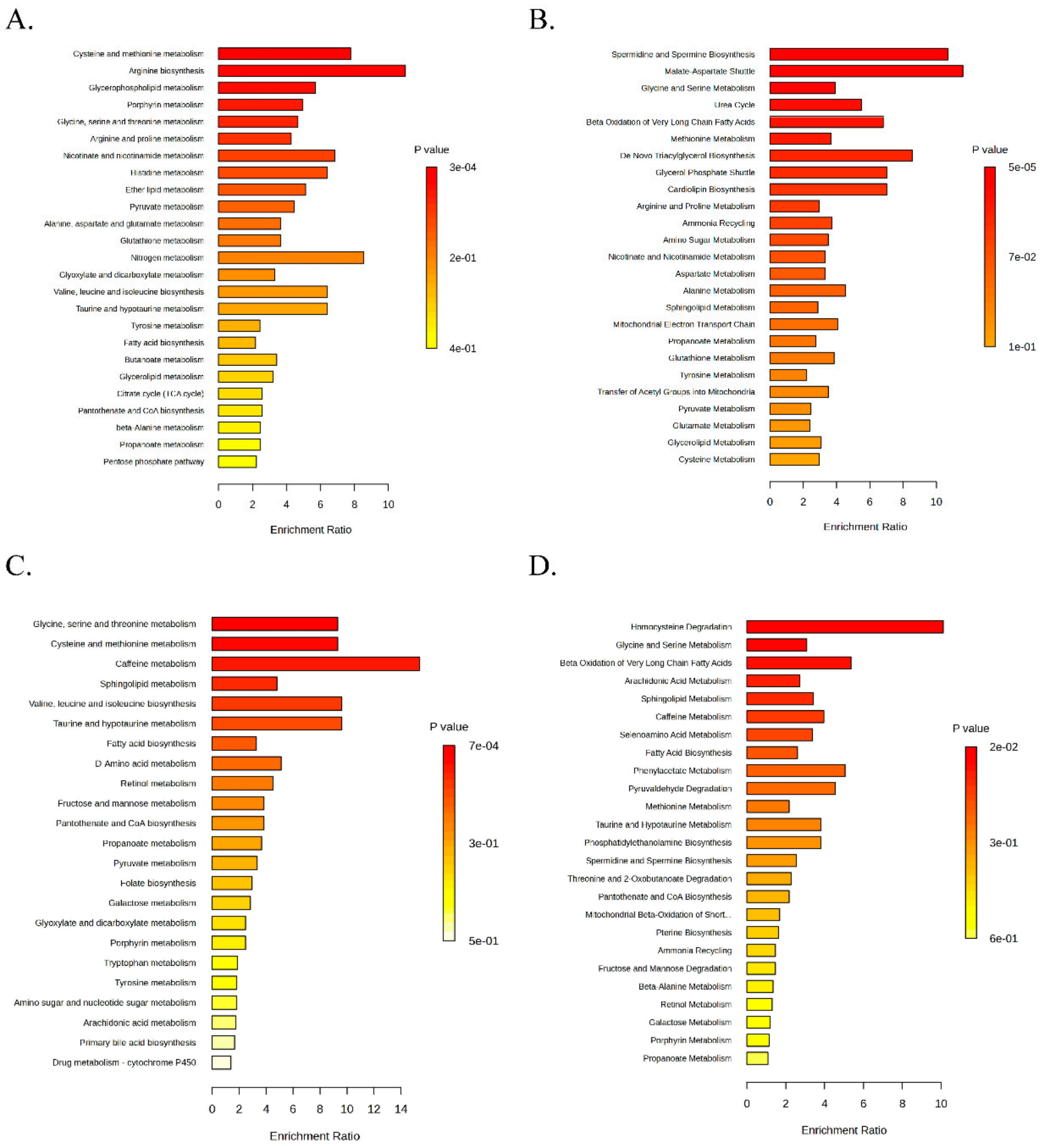

Figure S4. Enrichment Analysis of Differentially Expressed Genes for Silicosis Subtypes.

(A) KEGG enrichment for NMF1. (B) SMPDB enrichment for NMF1. (C) KEGG enrichment for NMF2. (D) SMPDB enrichment for NMF2.

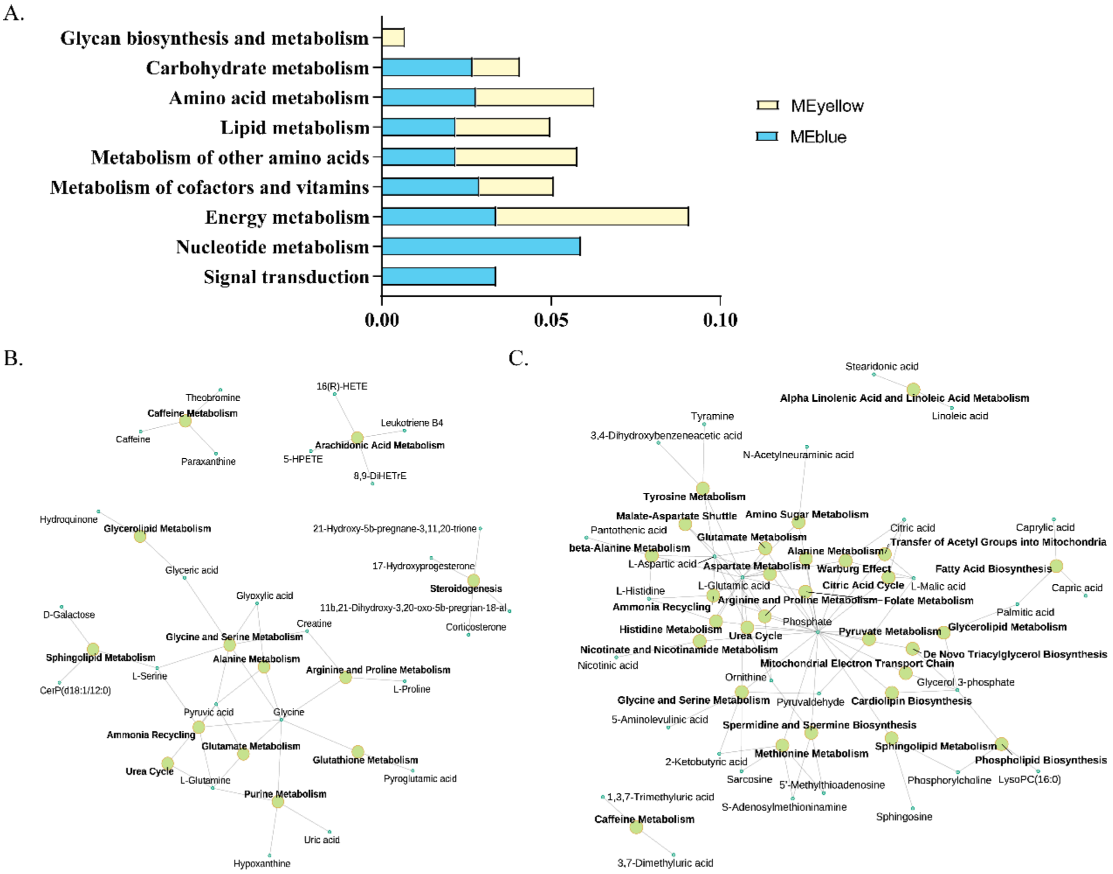

Figure S5. Metabolite Type Distribution and Regulatory Networks for Key Metabolites in Silicosis Subtypes.

(A) Distribution of MEblue and MEyellow modules across twelve major metabolite types. (B) Metabolic regulatory network for NMF1 subtype. (C) Metabolic regulatory network for NMF2 subtype.

Table S4. Expression Levels of 27 Selected Metabolites Across Silicosis Subtypes and Healthy Controls

metabolites	Control	NMF1	NMF2
Indoleacetic acid	2.54	1.40	1.25
N-Methylpropionamide	2.00	0.78	1.00
8-iso-15-keto-PGE2	5.14	5.40	5.78
Ethylphosphonic acid	0.46	0.34	0.26
Cafestol acetate	3.58	8.00	5.04
Spiroxamine	3.19	3.49	2.78
Drofenine	2.53	3.55	2.31
Palmitic acid alkyne	5.47	11.05	10.16
Anofinic_acid	2.41	0.89	0.69
2-(.beta.-D-Glucopyranosyloxy)benzyl 3-(hexopyranosyloxy)-6-hydroxy-2-methoxybenzoate	5.29	3.16	3.79
IRIGENIN	3.62	3.57	2.50
[(E)-4-Acetyloxy-5-(3,6-diacetyloxy-5,5,8a-trimethyl-2-methylidene-4-oxo-4a,6,7,8-tetrahydro-1H-naphthalen-1-yl)-3-methylpent-2-enyl] acetate	3.99	3.36	2.26
Acetylvalerenolic_acid	2.71	6.06	1.75
(R)-4-((7R,8S,9S,10R,13R,14S,17R)-7-hydroxy-10,13-dimethyl-3-oxo-2,3,6,7,8,9,10,11,12,13,14,15,16,17-tetradecahydro-1H-cyclopenta[a]phenanthren-17-yl)pentanoic acid	1.65	1.45	1.43
Annoglabasin_F	4.13	1.19	1.78
Solacauline	3.29	1.53	1.89
Arachidonyl trifluoromethyl ketone	5.60	1.25	2.21
2-[(2R,3S,7R,8R,8aS)-2,3,4'-Trihydroxy-4,4,7,8a-tetramethyl-6'-oxospiro[2,3,4a,5,6,7-hexahydro-1H-naphthalene-8,2'-3,8-dihydrofuro[2,3-e]isoindole]-7'-yl]-3-methylpentanoic acid	8.38	1.23	3.75
7-Hydroxycoumarin	8.44	10.63	7.72
Diethanolamine	2.16	0.67	0.96
Betaxolol	2.89	6.81	8.13
PI(20:4(5Z,8Z,11Z,14Z)/0:0)	2.82	2.31	3.03
Lovastatin hydroxy acid	15.05	13.66	9.66
Tyrosocholic acid	2.42	5.91	2.10
Enalaprilat	3.06	3.58	3.24
Benzenehexanamide, .beta.-hydroxy-N-[1-(hydroxymethyl)-3-methylbutyl]-.delta.-oxo-	4.03	11.40	12.42
Boviquinone_4	1.37	0.92	1.16
